# Treatment of velopharyngeal insufficiency in a patient with a submucous cleft palate using a speech aid: the more treatment options, the better the treatment results

**DOI:** 10.1186/s40902-019-0202-8

**Published:** 2019-05-01

**Authors:** Yun-Ha Park, Hyun-Jun Jo, In-Seok Hong, Dae-Ho Leem, Jin-A Baek, Seung-O Ko

**Affiliations:** 1 Department of Oral and Maxillofacial Surgery, School of Dentistry, Chonbuk National University Dental Hospital, 20, Geonji-ro, Deokjin-gu, Jeonju-si, Jeollabuk-do Republic of Korea; 2 Research Institute of Clinical Medicine-Biomedical Research Institute, Chonbuk National University Hospital, 20, Geonji-ro, Deokjin-gu, Jeonju-si, Jeollabuk-do Republic of Korea

**Keywords:** Speech aid, Velopharyngeal insufficiency, Submucosal cleft, Hypernasality

## Abstract

**Background:**

The submucous cleft palate (SMCP) is a type of cleft palate that may result in velopharyngeal insufficiency (VPI). Palate muscles completely separate oral and nasal cavities by closing off the velopharynx during functional processes such as speech or swallow. Also, hypernasality may arise from anatomical or neurological abnormalities in these functions. Treatments of this issue involve a combination of surgical intervention, speech aid, and speech therapy. This case report demonstrates successfully treated VPI resulted from SMCP without any surgical intervention but solely with speech aid appliance and speech therapy.

**Case presentation:**

A 13-year-old female patient with a speech disorder from velopharyngeal insufficiency that was caused by a submucous cleft palate visited to our OMFS clinic. In the intraoral examination, the patient had a short soft palate and bifid uvula. And the muscles in the palate did not contract properly during oral speech. She had no surgical history such as primary palatoplasty or pharyngoplasty except for tonsillectomy. And there were no other medical histories. Objective speech assessment using nasometer was performed. We diagnosed that the patient had a SMCP. The patient has shown a decrease in speech intelligibility, which resulted from hypernasality. We decided to treat the patient with speech aid (palatal lift) along with speech therapy. During the 7-month treatment, hypernasality measured by a nasometer decreased and speech intelligibility became normal.

**Conclusions:**

Surgery remains the first treatment option for patients with velopharyngeal insufficiencies from submucous cleft palates. However, there were few reports about objective speech evaluation pre- or post-operation. Moreover, there has been no report of non-surgical treatment in the recent studies. From this perspective, this report of objective improvement of speech intelligibility of VPI patient with SMCP by non-surgical treatment has a significant meaning. Speech aid can be considered as one of treatment options for management of SMCP.

## Background

The submucous cleft palate (SMCP), a type of cleft palate, is a congenital condition associated with abnormal development in muscle tissue of the soft palate [1]. It is characterized not by either a complete or incomplete cleft palate but by the disconnected muscle tissue and unbroken lining only in the middle of the soft palate [2].

These SMCP patients may present hypernasal speech from velopharyngeal insufficiency (VPI) as well as secretory otitis media and hearing loss from the malfunction of the Eustachian tube. Hypernasality is the most common symptom in VPI which accounts for about 50% of the SMCP patient cases [3].

Nasal and oral cavities are completely separated from one another during speaking, swallowing, blowing, and vomiting by closing off velopharynx. Velopharyngeal closure is a particularly important part in producing pressure-sensitive sounds [4].

However, abnormal muscle development within the soft palate as in SMCP patients often leads to abnormal velopharyngeal closure, which results in mispronunciation in speech including hypernasality.

Traditionally, in order to treat VPI in this SMCP patient, surgical intervention is the first treatment choice. If the patient shows remaining VPI after surgery, speech aid combined with speech therapy can be applied as an adjunctive method [2, 4]. There is no report of speech aid treatment as first choice in the management of SMCP patients.

This case reports a 13-year-old female patient with speech disorder caused by SMCP who showed significant objective improvements in speech from using palatal lift and speech therapy without any surgical intervention.

## Case presentation

### Subject

**Fig. 1:**
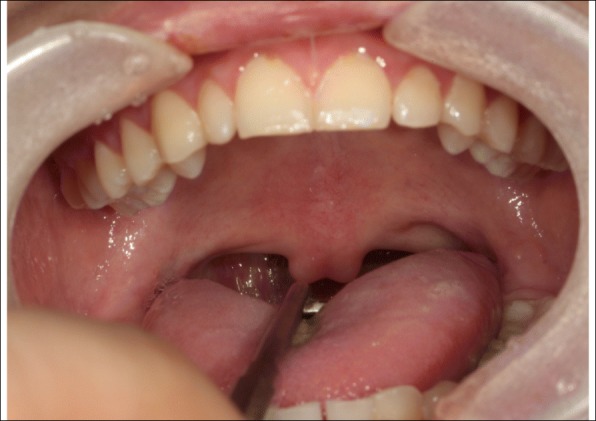
Intraoral view in rest position. The patient had a bifid uvula and short soft palate

**Fig. 2:**
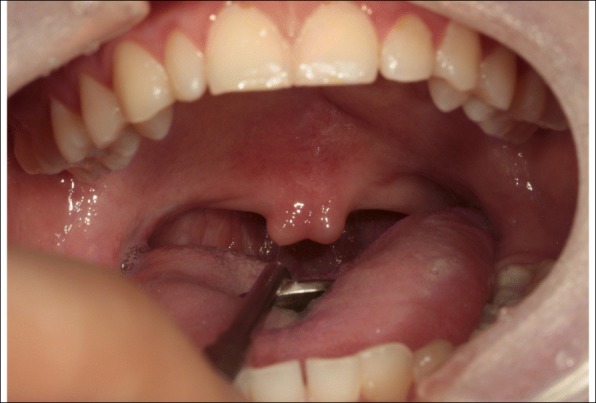
Intraoral view during pronouncing /a/. The VP function is not working well during pronunciation showing VPI. This patient showed severe nasal sound

A 13**-**year-old female patient visited the outpatient oral and maxillofacial department of this hospital due to speech problems. In the intraoral examination, the patient had a short soft palate and bifid uvula. Also, the movement of the soft palate was very limited during speech (Figs. 1 and 2). An objective assessment was conducted. The patient showed severe hypernasality, articulation disorder, and low speech intelligibility. She did not show language retardation.

Upon these signs, the patient was diagnosed with a VPI with SMCP, and we decided to provide palatal lift treatment and speech therapy.

### Speech assessment

#### Device

##### Nasometer II 6400 (USA)

Higher nasalance shows higher waveform, and nasalance percentages less than or equal to 20% are considered to represent the absence of nasality and are marked with a green line [5].

#### Vocal language samples

1. Sustained phonation
  - Single vowel: /a/,/i/,/e/,/u/
  - Double vowel: /ja/,/je/,/wi/
2. Syllable repetition
  - Bilabial plosive: /papi./, /phaphi/, /p’ap’i/
  - Alveolar plosive: /tati/, /thathi/, /t’at’i/
  - Velar plosive: /kaki/, /khakhi/, /k’ak’i/
  - Aveolopalatal fricative: /cica/, /chicha/, /c’ic’a/
3. Sentence (nasal consonant ratio, NCR 0%): /wɔljoil ohu patatkae kasɔ cokε sɛulɯl cabko hwajoil sεpjɔke tolaoketta/

#### Results of assessment

An assessment of nasalance prior to treatment showed severe nasalance in high vowels /i/,/wi/, high nasalance in /e/,/o/,/u/,/je/, and mild nasalance in /a/,/ja/, as well as hypernasality in syllable repetition and sentences (Table 1).

**Table 1:** Results of nasometric assessment of the patient before intervention

	Nasalance score (%)												
	Vowels								Syllable repetition				Sentence
	/a/	/i/	/e/	/o/	/u/	/ja/	/je/	/wi/	/papi/	/tati/	/kaki/	/cica/	/~patakae~/*
Normal (%)	8.6	22.3	8.7	8.4	10	8.5	8.6	20.5	16.9	18.6	21.1	16.9	11.1
Patient (Before Treatment)	46	88	58	59	61	44	60	83	71	68	75	74	58

*/wɔljoil ohu patatkae kasɔ cokɛ sɛulɯl cabko hwajoil sɛpjɔke tolaoketta/

### Treatment plan

Her parents did not want surgical treatment. We offered the pilot study of conservative treatment using speech aid/speech therapy without surgery, and they accepted the treatment method. The patient showed the closure failure of the velopharyngeal port due to the short soft palate and insufficient contraction of the palatal muscles. Therefore, a palatal lift which elevates the soft palate superiorly was selected. We educated the patient that the palatal lift should be fitted for all hours except when sleeping. The assessment was scheduled to be performed once a month, and weekly speech therapy was recommended. However, the patient was only able to come in once or twice a month.

**Fig. 3:**
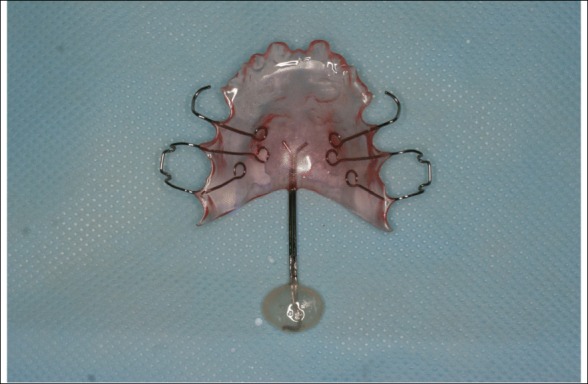
Palatal lift: the functional part can elevate the soft palate

Palatal lift was made and applied to the patient (Fig. 3). Speech therapy including visual feedback, muscle training, perception training, and speech assessment using nasometer was performed at each visit.

## Results

**Fig. 4:**
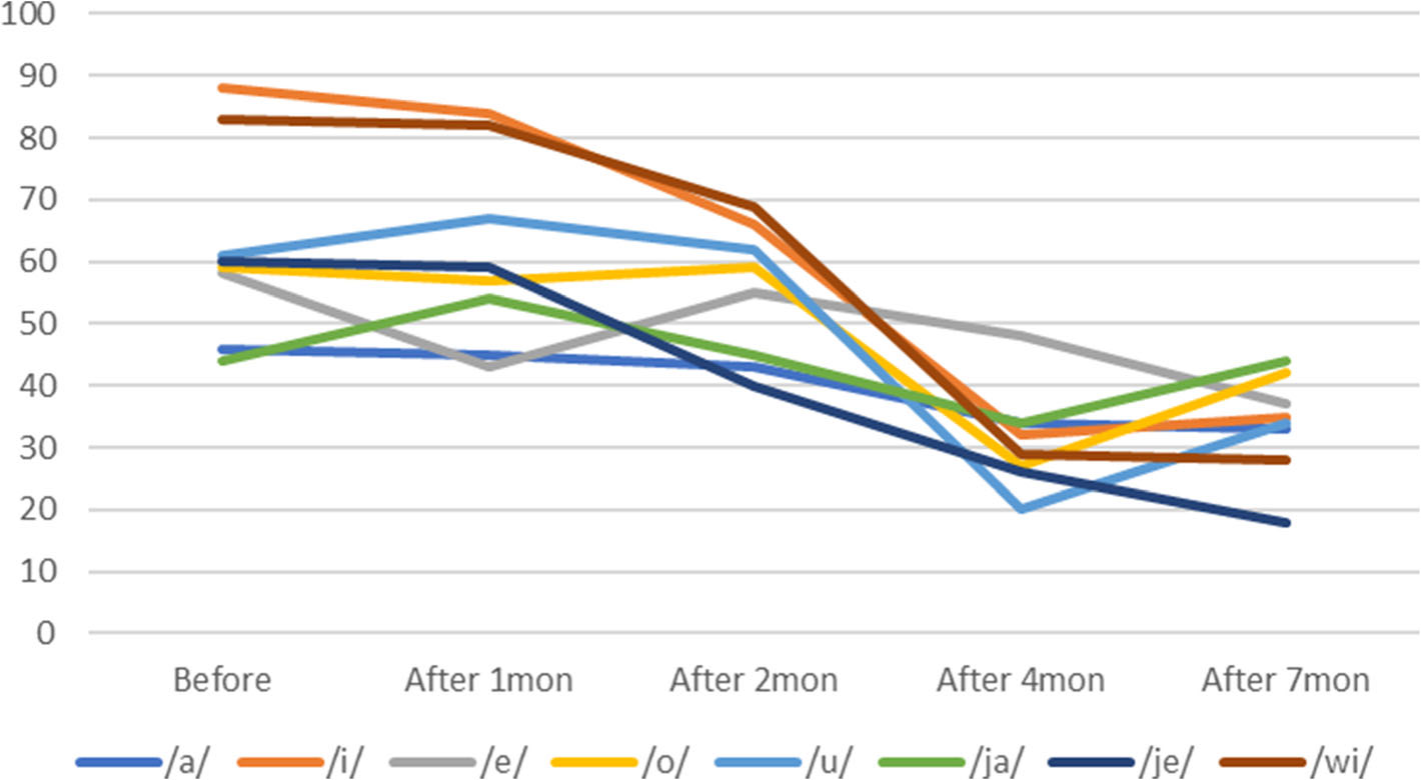
Evaluation of hypernasality (vowels). Measurements of nasal emission energy in vowels using nasometer. After initiation of speech aid therapy, the nasality decreases with time

**Fig. 5:**
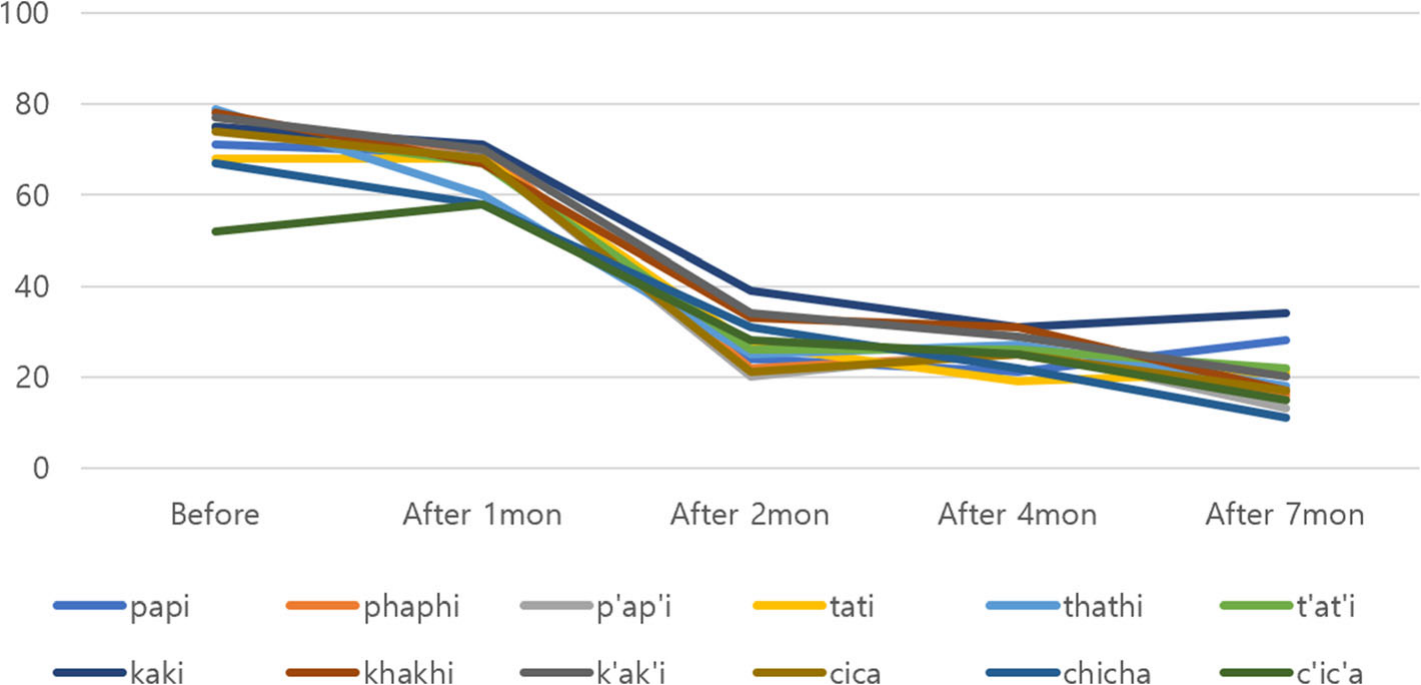
Evaluation of hypernasality (syllable repetition). Measurements of nasal emission energy in syllable repetition using nasometer. After initiation of speech aid therapy, the nasality decreases with time

**Fig. 6:**
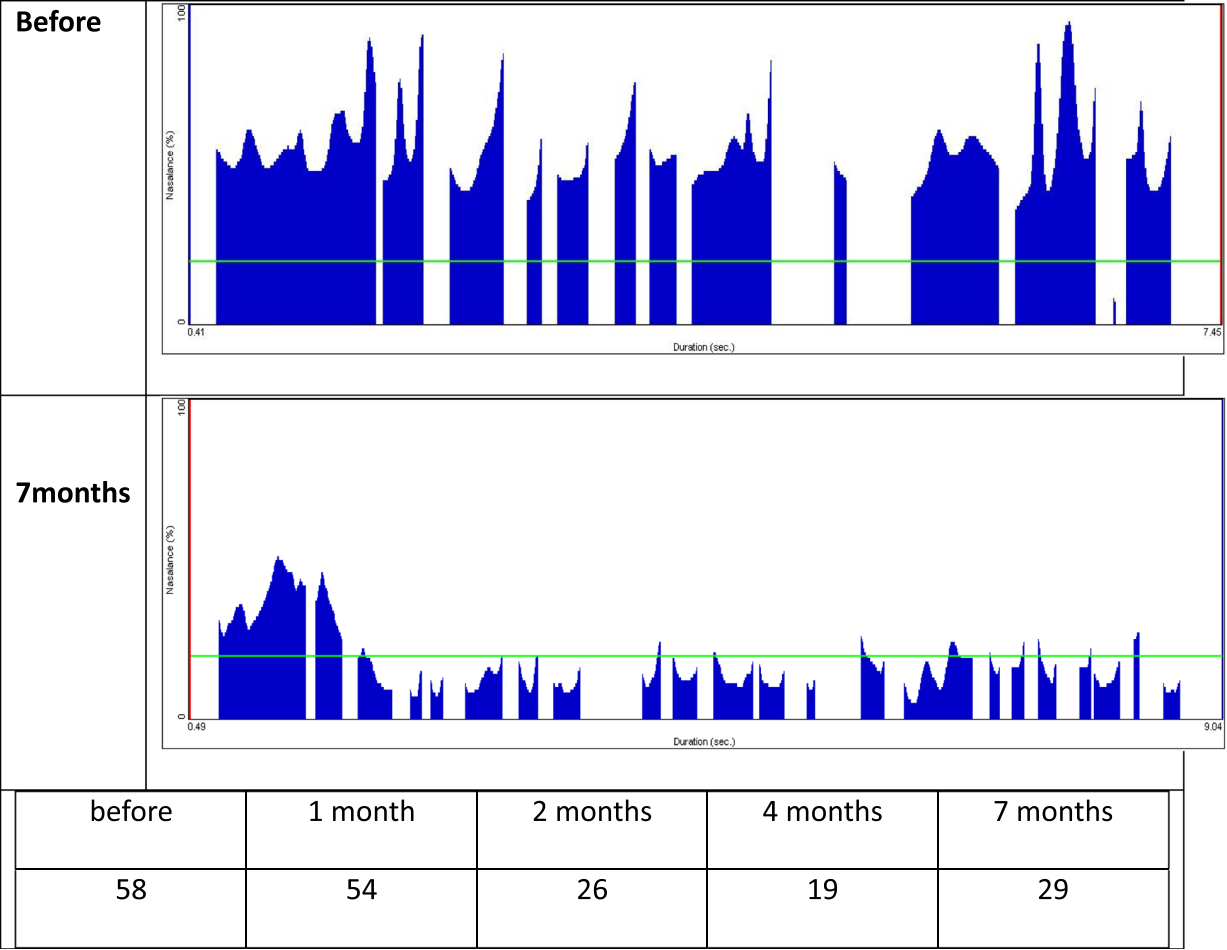
Evaluation of hypernasality (sentence). Measurements of nasal emission energy in “nasal consonant ratio (NCR) 0% sentence” using nasometer. Nasalance percentages less than or equal to 20% are considered to represent the absence of nasality and are marked with a green line. The nasality was significantly decreased when compared with 7 months after installation

The articulation of the single vowel /i/ improved the most after treatment, from 88% prior to treatment. Although each vowel presented different reductions in vowel pronunciation, all vowels showed decreased hypernasality (Fig. 4). While nasalance in syllable repetition indicated hypernasality in all pronunciations prior to treatment, it gradually decreased to the normal nasalance range as treatment progressed (Fig. 5). When the patient pronounced the sentence with an NCR of 0%, the amount of energy that was delivered to nasal cavity averaged 54% prior to speech aid therapy and was identified to average 29% after 7 months, which was close to the normal nasalance of 20%. Less than 20% of nasalance was also observed in most sentence pronunciations (Fig. 6). Based on these results, we concluded that the hypernasality, which was a major symptom of the patient, was successfully improved.

At 7 months after the treatment began, the patient showed normal nasalance and speech intelligibility without nasal emission, and we started the time-based reduction program at 10 months. The time-based reduction program is to gradually reduce a size of the functional part and/or an applied time of the appliance.

## Discussion

Calnan [6] defined SMCP as the imperfect union of muscles that cross the soft palate, and patients with SMCP have shown soft palate shortness and velopharyngeal closure, which resulted in hypernasality and unintelligible speech [2]. This report established Calnan’s triad as criteria for diagnosis SMCP, which included bifid uvula, clear lining in the middle of the soft palate, and the absence of a bony notch in the posterior margin of the hard palate, as found in patient with SMCP [7]. However, not all patients with SMCP show this triad of signs; some patients present only one or two signs.

Also, cleft palate muscle malposition may occur in the absence of the triad signs, which is a condition that has been identified as occult submucous cleft palate (OSCP). OSCP is difficult to recognize by oral examination alone but can be confirmed during surgery [2, 8].

The major symptoms of SMCP are hypernasality (51%) due to motile incompetence in the soft palate and pharyngeal muscles, as well as conductive hearing loss (45%) [3, 9]. Palatoplasty is performed to connect palate muscles to stretch the length of the soft palate. However, the postoperative frequency of VPI has been reported to range from 20 to 50%, even with successful surgery [10]. Accordingly, it is necessary to proceed with prosthetic treatment using a combination of speech aid and speech therapy to provide an ideal treatment [9].

Sphincteric interaction of the palate in the pharynx is very important for producing intelligible speech [10]. Speech problems that include hypernasality may arise when these palate muscles function inappropriately. There are mainly three muscles that affect velopharyngeal closure. First, during the contraction of levator veli palatini muscles, the soft palate is lifted and conveyed into the posterior direction. Superior pharyngeal constrictor muscles move to constrict the pharyngeal cavity in a circular shape by moving forward and to the inside of the pharynx’s lateral wall. Last, uvula muscles provide thickness at the posterior position [4].

Velopharyngeal dysfunction (VPD) is a general term that describes an inappropriate function of velopharyngeal port [11]. Velopharyngeal insufficiency (VPI) is a congenital or acquired condition in which the velopharynx has not closed due to a lack of soft tissue. The most common cause of VPI is cleft palate, including SMCP and occult submucous cleft palate. These patients present hypernasality, nasal emission, and reduced speech intelligibility [4]. Velopharyngeal incompetence refers to a functional velopharyngeal impairment that is due to neuromuscular diseases, such as cerebral palsy, myotonic dystrophy, and cerebral vascular accidents. Also, velopharyngeal mislearning (VPM) indicates speech problems with learning a language, which are not caused from anatomical or neurophysiological reasons [12].

In order to treat velopharyngeal insufficiency and incompetence, surgical intervention should be considered combined with prosthetic treatment and speech therapy. And surgical intervention is the first line of treatment. Although there are various surgical procedures, the operation has a success rate of about 50%. Speech aids can be a good alternative when surgical treatment is not considered.

Speech aid consists of the maxillary portion (palatal portion) covering the palate, the pharyngeal portion (functional part), and the palatal extension that connects between of them. Speech aids can be divided into speech bulbs and palatal lift depending on the functional part [2]. The functional part of speech bulb directly closes the opened velopharyngeal port during pronouncing [13]. The functional part of palatal lift is placed on the soft palate and elevates the soft palate to the posterosuperior position [12].

During treatment with speech aids, periodic speech therapy and assessment are essential, and if the nasality and speech intelligibility of the patients are normal without nasal emission, time-based reduction program of the functional part can be initiated [11]. Finally, when the patient sounds the same whether the appliance is worn or removed, the appliance may then be removed permanently.

After reduction therapy, if the appliance cannot be reduced additionally or removed entirely, an appropriate surgical procedure may then replace it.

In this case, the patient showed much progress in nasalance and speech intelligibility using palatal lift and speech therapy without any surgical intervention. It suggests that conservative treatment without surgical intervention may be an effective treatment for SMCP patients with VPI.

## Conclusion

About 50% of patients with submucous cleft palates suffer from speech problem due to velopharyngeal insufficiencies. Surgical intervention is the first treatment option to be considered. However, this case shows that speech aid without any help of surgical management can be another treatment option for speech problem caused by SMCP.

Having a variety of treatment strategies is helpful to establish a more suitable treatment plan. Speech aid can be a viable treatment option to replace surgical intervention in patients with SMCP. There are only few studies on the non-surgical treatment of the SMCP patients, and further studies should be continued in this field.

## Abbreviations

NCR: Nasal consonant ratio
SMCP: Submucous cleft palate
VPD: Velopharyngeal dysfunction
VPI: Velopharyngeal insufficiency
VPM: Velopharyngeal mislearning
